# H3K27M neoepitope vaccination in diffuse midline glioma induces B and T cell responses across diverse HLA loci of a recovered patient

**DOI:** 10.1126/sciadv.adi9091

**Published:** 2024-02-02

**Authors:** Tamara Boschert, Kristina Kromer, Taga Lerner, Katharina Lindner, Gordon Haltenhof, Chin Leng Tan, Kristine Jähne, Isabel Poschke, Lukas Bunse, Philipp Eisele, Niklas Grassl, Iris Mildenberger, Katharina Sahm, Michael Platten, John M. Lindner, Edward W. Green

**Affiliations:** ^1^ CCU Neuroimmunology and Brain Tumor Immunology, German Cancer Research Center (DKFZ), Heidelberg, Germany.; ^2^ Faculty of Biosciences, Heidelberg University, Heidelberg, Germany.; ^3^ Helmholtz Institute for Translational Oncology (HI-TRON Mainz) - A Helmholtz Institute of the DKFZ, Mainz, Germany.; ^4^ BioMed X GmbH, Heidelberg, Germany.; ^5^ Immune Monitoring Unit, DKFZ and National Center for Tumour Diseases (NCT), Heidelberg, Germany.; ^6^ Department of Neurology, Medical Faculty Mannheim, MCTN Heidelberg University, Mannheim, Germany.; ^7^ DKFZ Hector Cancer Institute at the University Medical Center Mannheim, Mannheim Germany.

## Abstract

H3K27M, a driver mutation with T and B cell neoepitope characteristics, defines an aggressive subtype of diffuse glioma with poor survival. We functionally dissect the immune response of one patient treated with an H3K27M peptide vaccine who subsequently entered complete remission. The vaccine robustly expanded class II human leukocyte antigen (HLA)–restricted peripheral H3K27M-specific T cells. Using functional assays, we characterized 34 clonally unique H3K27M-reactive T cell receptors and identified critical, conserved motifs in their complementarity-determining region 3 regions. Using detailed HLA mapping, we further demonstrate that diverse HLA-DQ and HLA-DR alleles present immunogenic H3K27M epitopes. Furthermore, we identified and profiled H3K27M-reactive B cell receptors from activated B cells in the cerebrospinal fluid. Our results uncover the breadth of the adaptive immune response against a shared clonal neoantigen across multiple HLA allelotypes and support the use of class II–restricted peptide vaccines to stimulate tumor-specific T and B cells harboring receptors with therapeutic potential.

## Teaser

Adaptive immune responses are functionally characterized in a diffuse midline glioma patient in remission upon H3vac treatment.

## INTRODUCTION

A recurrent monoallelic, nonsynonymous mutation at amino acid position 27 in the genes encoding histone-3.3 or 3.1 (H3K27M) defines a distinct subtype of highly aggressive diffuse midline glioma (DMG) characterized by high morbidity and universal lethality, with a median overall survival of 11 months after diagnosis (*1*–*3*). Because of infiltrative growth in midline brain structures and resistance to chemotherapy (*4*), radiotherapy remains the main effective treatment option for DMG (*5*–*7*)*.* Because of a low mutational burden and a lack of immune cell infiltration, DMG is generally nonresponsive to immune checkpoint inhibition treatment but may benefit from personalized treatment or targeted immunotherapies (*5*–*7*). A recent clinical trial of chimeric antigen receptor T cell therapy targeting the uniformly upregulated disialoganglioside GD2 in DMG (*8*, *9*) showed a radiographic improvement and reduction of clinical symptoms in three of four patients (*9*), and additional trials targeting other antigens are ongoing (*10*–*12*).

The recurrent clonal neoepitope H3K27M represents an attractive intracellular target for immunotherapy. We and others have demonstrated presentation of H3K27M-derived epitopes on class I and class II human leukocyte antigen [HLA, the human nomenclature for the major histocompatibility complex (MHC)] molecules capable of stimulating mutation-specific CD4^+^ and CD8^+^ T cell responses both in MHC humanized mice and in healthy human donors. Together, these studies have shown that HLA-DRB1*01:01–restricted CD4^+^ T cells and HLA-A*02:01–restricted CD8^+^ T cells induced by therapeutic long (H3K27M_p14–40_) or short (H3K27M_p26–35_) peptide vaccines, respectively, display antitumor efficacy in vitro and in vivo (*13*–*15*). While efficient endogenous presentation of the short epitope on HLA-A*02:01 is controversial (*14*–*16*)*,* a recently published phase 1 trial demonstrated that patients with H3K27M-specific CD8^+^ immunological responses to a short-peptide vaccine had prolonged overall survival relative to nonresponders (*15*).

We and others have used long-peptide vaccines targeting neoepitopes to stimulate antitumor T helper cell responses in early clinical trials (*17*–*19*); however, the breadth of the T cell response is not well understood. Class II HLA–restricted epitopes can induce CD4^+^ T cell responses in patients with diverse *HLA* allelotypes (*17*–*19*), but the precise restriction has not been assessed, mainly because of the lack of appropriate tools for high-throughput testing. Similarly, while B cell contributions have been described following long-peptide vaccine administration, the quality of the antibody responses has not been determined (*17*).

We recently initiated a phase 1 first-in-human clinical trial testing the safety and efficacy of H3-vac, a long vaccine encompassing the (H3K27M_p14–40_) epitope in patients with newly diagnosed DMG (clinicaltrials.gov identifier NCT04808245). Here, we present an in-depth functional study identifying and characterizing H3K27M-specific CD4^+^ T cell receptors (TCRs) and B cell receptors (BCRs) from the blood and cerebrospinal fluid (CSF) of a patient (ID01) not eligible for the trial vaccinated on a compassionate use basis as part of a case series profiling safety and dose scheduling of H3-vac recently reporter by Grassl and colleagues (*20*). This patient exhibited a sustained clinical response to the vaccine, offering the unique opportunity to characterize biologically relevant immune responses. These efforts shed light on potential mechanisms driving the survival of exceptional patients and potentially offer new therapeutic modalities for diagnosed patients with H3K27M^+^ DMG.

## RESULTS

### H3-vac induces a CD4^+^ T cell–specific immune response

Patient ID01 was vaccinated with the long H3K27M_14–40_ peptide according to a predefined vaccination schedule consisting of four biweekly administrations, followed by four treatments at 1-month intervals, then ongoing quarterly immunizations. Longitudinal blood samples were subjected to detailed immunophenotyping, including enzyme-linked immunosorbent spot (ELISpot) assays and TCR repertoire sequencing.

**Fig. 1.**
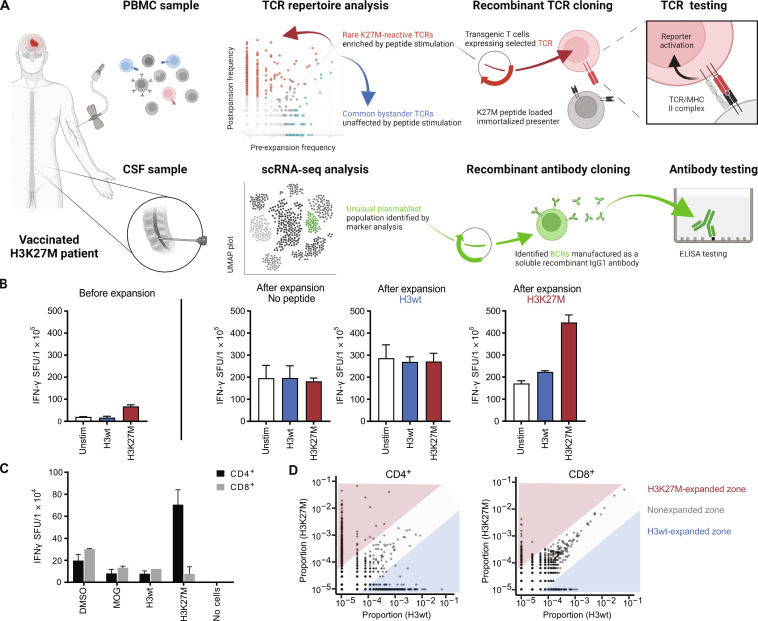
Identification and expansion of putative H3K27M-reactive TCRs. (**A**) Schematic of the TCR expansion, selection, and screening pipeline. Blood and CSF were collected regularly. T cells were expanded from PBMC using H3K27M peptide, H3wt peptide, or no peptide. Expansion analysis of clonotypes was performed using TCRβ deep sequencing, and clonotype frequency was plotted of H3K27M-expanded clones over H3wt-expanded clones. Single-cell sequencing was conducted for the PBMC expanded with H3K27M peptide and CSF samples. Most frequent TCRs and BCR identified by these two selection processes are cloned and functionally validated. (**B**) H3K27M-specific immune response assessed by IFN-γ ELISpot assay. IFN-γ spots are depicted for PBMC before expansion and after ex vivo restimulation with the respective peptide. Columns represent the means of technical triplicates. Technical duplicates in panel 4. (**C**) IFN-γ ELISpot assay of CD4^+^ and CD8^+^ T cells isolated from PBMC before expansion. SFU, spot-forming units. (**D**) Dot plots showing the frequency of H3K27M- over H3wt-expanded clonotypes in the CD4^+^ (left) and CD8^+^ T (right) cell population after expansion. Schematics were created using BioRender.com.

Following partial resection to remove the DMG from the right thalamus and standard radiotherapy in combination with temozolomide 11 days before the initial vaccination, patient ID01 showed an initial radiographic pseudoprogression followed by complete remission extending more than 3 years after diagnosis (fig. S1A). The patient displayed a strong H3K27M vaccine–induced immune response (relative to the prevaccination baseline) at 4 weeks after vaccination (fig. S1, B and C). Ex vivo expansion of peripheral blood mononuclear cells (PBMCs) from this time point in the presence of H3wt or H3K27M peptide (Fig. 1A) resulted in a general increase in interferon-γ (IFN-γ) release; however, a specific increase of IFN-γ–secreting cells was only observed in samples expanded and restimulated with the mutant H3K27M peptide (Fig. 1B). ELISpot assays with isolated CD4^+^ and CD8^+^ T cells from postvaccination PBMC (before ex vivo expansion) showed that this effect is predominantly driven by CD4^+^ T cells (Fig. 1C). In addition, the presence of a class II HLA–blocking antibody significantly reduced the number of IFN-γ–producing, H3K27M-specific cells, while class I HLA-blocking did not suppress the in vitro epitope response (fig. S1D).

Following peptide expansion, PBMCs were sorted into CD4^+^ and CD8^+^ fractions and each was subjected to TCR-β chain repertoire sequencing to assess the expansion dynamics of individual TCR clonotypes. We observed that individual CD4^+^ T cell clonotype frequencies shifted markedly in response to mutant or wild-type H3 peptide stimulation, while CD8^+^ T cell TCR clonotypes expanded nonspecifically (Fig. 1D), further suggesting that the vaccine-induced immune response was driven by CD4^+^ T cells.

### H3-vac expands a polyclonal H3K27M-specific TCR repertoire

**Fig. 2.**
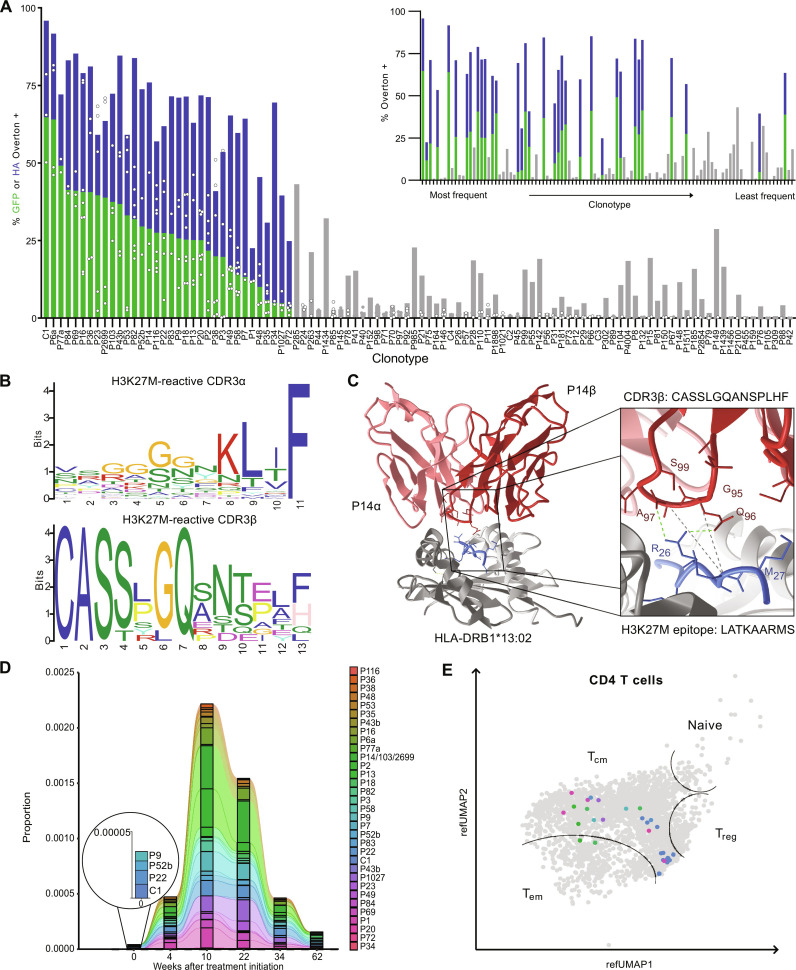
Validating the H3K27M reactivity of patient ID01 TCRs in a Jurkat reporter line. TCR nomenclature in figures reflects their origin and frequency, P indicates that the TCR was identified first from expanded PBMC and C from the CSF, with the index indicating their frequency rank. The lower the number, the more frequent the TCR. If a β chain paired with two α chains shares the same frequency, this is reflected by TCRs named with the same clonotype number followed by a or b, e.g., P6a/ P6b. (**A**) Superimposed dual reporter signals from GFP (green) and HA staining (blue) after coculture of transduced reporter cells and ID01 B cell line with 10 μM H3K27M peptide. The signal is determined by flow cytometry using Overton positive referenced to H3wt-treated samples. TCRs are arranged by GFP signal (main plot) or clonotype frequency (upper right corner). Gray TCRs do not pass criteria to be considered reactive (fig. S3B). (**B**) Amino acid motif of CDR3 α (top) and β (bottom) chains of all reactive TCRs. Motif analysis was performed using XSTREME tool. Shuffled input sequences and TCRβ deep sequencing data of the baseline repertoire were used as control sequences for finding the CDR3α and CDR3β motif, respectively. (**C**) High-resolution modeling of complete pMHC:TCR complex (left) of H3K27M-reactive TCR P14. Interacting bonds of the CDR3β (red) residues with the peptide (blue) are indicated in the magnified panel. (**D**) Longitudinal TCRb deep sequencing was performed to track the proportion of H3K27M-reactive clonotypes in peripheral blood at the indicated time points. (**E**) UMAP of CD4^+^ T cell cluster indicating T cell identity of reactive TCR clonotypes (right) from CSF aspirates 34 weeks after treatment initiation.

To confirm that individual TCRs recognize H3K27M epitopes, peptide-expanded PBMC were submitted for single-cell VDJ (scVDJ) sequencing to determine α/β chain pairing and reconstitute full TCR clonotypes. Given that H3K27M-reactive TCRs effecting and/or orchestrating brain tumor clearance would be enriched in the CSF, we also performed scVDJ sequencing on cells from post-treatment CSF samples. We synthesized and cloned a total of 102 TCRs, giving preference to clonotypes with the greatest ex vivo expansion, with the expectation that these were most likely to be H3K27M specific. TCR clonotypes expressing two α chains were screened separately but considered to be representative of a single bona fide TCR clone (Fig. 2A and fig. S2D).

TCRs were screened for reactivity against H3K27M using the T-FINDER platform (*21*). Briefly, patient-derived B cells were immortalized to generate a fully autologous and HLA-competent B lymphoblastoid cell line (B-LCL), while Jurkat cells integrated with a highly sensitive dual reporter of T cell activation were transgenically modified to express each TCR of interest (fig. S2A). The B-LCLs were pulsed with H3wt or H3K27M peptides and cocultured for 16 hours with each transgenic TCR reporter cell line. Upon cognate TCR:peptide interactions, the reporter produces a T cell–intrinsic green fluorescence protein (GFP) signal and marks cocultured B-LCLs by secreting a hemagglutinin (HA)–tagged αCD19 scFv, both of which can be assessed by flow cytometry.

Of the 102 TCRs from patient ID01 that were tested, 34 reacted specifically to the H3K27M mutant peptide but not the wild-type control (Fig. 2A). Although several less frequent TCRs also showed H3K27M reactivity, those with the strongest ex vivo expansion were indeed enriched for reactive clonotypes (Fig. 2A, inset). For clonotypes natively expressing two α chains, in all cases only a single α chain was reactive, demonstrating that binding of the H3K27M epitope required contributions from both chains (fig. S2E).

TCRs varied markedly in their response to H3K27M; we assigned a series of conservative thresholds to eliminate false positives, e.g., excluding TCRs with cross-reactivity to H3wt (see fig. S2B for further details). Specific H3K27M TCR activities ranged from 64.8% GFP positive / 95.9% HA positive to 3.1% GFP positive / 24.9% HA positive. The TCR with the greatest activity in our coculture assay was also the most prevalent TCR in the CSF (C1); the low frequency of this sequence before vaccination is indicative of strong clonal expansion and selection in vivo (Fig. 2D). Notably, this TCR also activates T cells in response to presentation of the vaccinating peptide by the tumor cell line U87-MG (fig. S2F), which has the autologous class-II HLA allele for C1 and naturally expresses considerable amounts of surface HLA-DQ. Overall, patient ID01’s tumor-specific TCR repertoire was highly polyclonal (fig. S2C), though we did observe evidence of convergent complementarity-determining region 3 (CDR3) selection: TCRs P14, P103, and P2699 all share the same CDR3α and CDR3β amino acid sequence but differ at their recombination junction site nucleotide sequences (fig. S2D). This phenomenon has previously been associated with antigen-specific T cell responses to cancer immunotherapies (*22*).

Relative to baseline, a longitudinal analysis of *TRBV* and *TRBJ* gene segment usage across the entire PBMC TCR repertoire shows no changes between visits (fig. S3A). However, the presence of *TRBV7–2* in patient ID01 is significantly overrepresented in the reactive TCRs relative to the baseline TCR repertoire (Fisher’s exact test, *P* < 0.005) (fig. S3B). In addition, we analyzed the recombination and usage of *TRBD* gene segments after observing the potential overrepresentation of a central glutamine residue in the CDR3β regions of reactive TCRs. The human *TRBD* locus contains two gene segments that can be inserted directly or inversely and read in three translational frames; only one combination of gene segment selection, orientation, and reading frame encodes this glutamine (Q) residue. Relative to the baseline TCR repertoire, the appearance of glutamine among reactive TCRs is, indeed, statistically enriched (Fisher’s exact test, *P* < 0.00005) (Fig. 2B). This glutamine residue forms the core of an enriched sequence motif present in the CDR3β of eight peptide-reactive TCRs, which is frequently observed in combination with a significantly enriched GGx_1-3_K motif in cognate TCR CDR3α sequences (Fig. 2B). To better understand the relevance of these motifs, we applied structural TCR-pMHC modeling using ImmuneScape (*23*). While structural and/or dynamic modeling studies are required to fully characterize these interactions, the CDR3β-conserved glutamine appears to interact directly with R_26_ of the H3K27M peptide in a model of the P14 TCR in complex with the peptide LATKAARMS presented by HLA-DRB1*13:02 (Fig. 2C; see the next section for TCR restriction mapping).

Cataloging this set of functionally validated H3K27M peptide–reactive TCRs enabled a focused reanalysis of the longitudinal TCR sequencing datasets. While reactive clonotypes could not be identified using TCR network analysis only, a post hoc analysis revealed significant expansion of H3K27M-reactive clones within the full repertoire for 10 weeks after administration of the first vaccine, followed by a decrease (Fig. 2D). At baseline, P52b, P9, P22, and C1 were detectable at very low proportions in bulk TCRβ sequencing. At 62 weeks after treatment initiation, the proportion of reactive TCRs remained higher than at baseline. At weeks 10 and 22, when the clonal expansion was at its greatest, the most frequent TCRs in the peripheral blood were the clonally converged P14/P103/P2699. At the final time point, these TCRs also comprise the greatest portion of reactive TCRs (Fig. 2D). A subset of H3K27M-reactive TCRs was also found in the CSF (Fig. 2E), including the convergently selected P14/P103/P2699 cluster, as well as P1, P2, P9, P18, P20, P22, P69, and P1027. We complemented our scVDJ with single-cell RNA (scRNA) sequencing and observed that these T cells almost exclusively exhibited a CD62L^+^CCR7^+^CD27^+^CD95^+^TCF7^+^ central memory phenotype (Fig. 2E).

### H3K27M epitopes are presented by both HLA-DR and HLA-DQ alleles in patient ID01

The diversity of HLA alleles which can present H3K27M-derived epitopes critically determines the number of patients that would benefit from therapeutic vaccination and/or TCR-T cell therapies. Computational algorithms such as NetMHCIIpan do not predict any strongly binding HLA:epitope complexes (*24*); therefore, we applied a two-step approach to empirically determine the HLA restriction (and thus the peptide-presenting allele) of each reactive TCR.

**Fig. 3.**
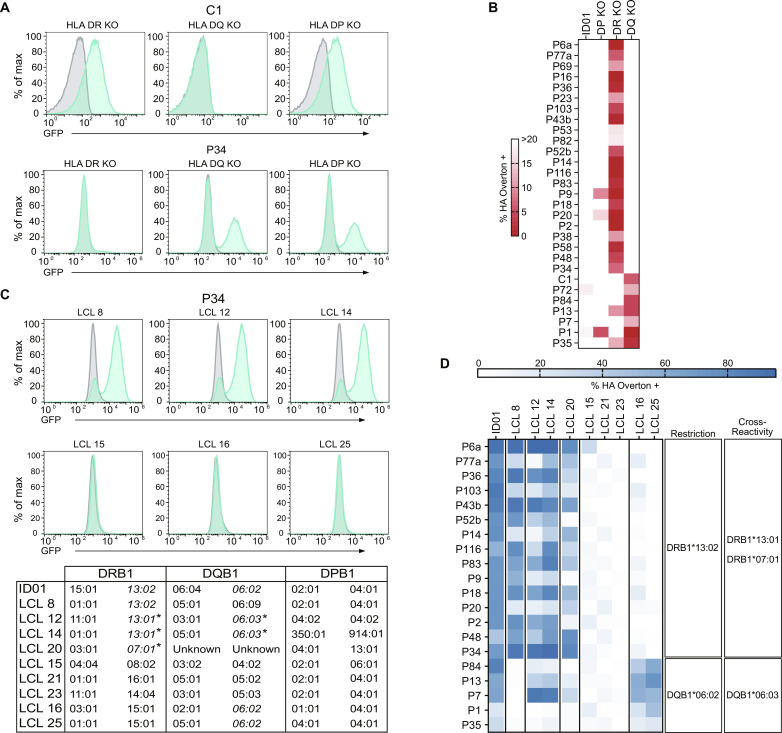
H3K27M HLA restriction analysis. (**A**) Representative flow cytometry histograms of H3K27M-reactive TCRs cocultured with peptide-pulsed autologous HLA class II knockout B-LCL (H3K27M in green and H3wt peptide in gray) depicting GFP reporter levels. (**B**) Heatmaps of H3K27M peptide–reactive TCRs cocultured with either autologous donor B-LCL or their corresponding ID01 HLA class II–deficient B-LCL. (**C**) Top: Representative histograms of HLA allele restriction screening against B-LCL from a healthy donor library. Bottom: *HLA* alleles of ID01 and LCL lines used in analysis. Cross-reactive *HLA* alleles are highlighted with an asterisk. (**D**) Restriction analysis screening heatmap of H3K27M-reactive TCRs from ID01. Percentage of HA-positive B-LCL was determined by the Overton method using H3wt peptide–pulsed controls for histogram subtraction.

First, we generated *HLA-DPA*, *HLA-DQA*, and *HLA-DRA* knockouts of patient ID01’s B-LCL (fig. S4A). The resulting cell lines were loaded with either H3K27M or H3wt peptide and cocultured with reactive TCR-transgenic reporter cells (Fig. 3, A to C, and fig. S2A). As expected, disrupting the restricting *HLA* locus abrogated TCR reactivity completely, with most clonotypes showing restriction to HLA-DR and several to HLA-DQ (Fig. 3, A and B).

In the second step, a panel of healthy donor B-LCL that share one or more *HLA* alleles with ID01 (Fig. 3C) was used to identify the restricting allele and screen for additional, cross-restricted *HLA* alleles. The healthy donor B-LCL were peptide pulsed and cocultured with reactive TCR-expressing T cells as above, and the combination of response-inducing lines was used to identify the presenting allele of each TCR (Fig. 3D). All ID01 HLA-DR–responsive TCRs were positive with LCL 8, LCL 12, and LCL 14, indicating they are restricted to DRB1*13:02, as well as the closely related nonautologous DRB1*13:01. Most of the HLA-DR–restricted TCRs also showed some response to peptide presentation by lines LCL 24 and LCL 20, indicating that the H3K27M peptide is presented by DRB1*07:01—contrary to HLA supertype predictions (*25*). The five HLA-DQ restricted TCRs were mapped to DQB1*06:02 (LCL lines 16 and 250), and with the exception of the weakly reactive TCR P1, also reacted with the DQB1*06:03-expressing LCL lines 12 and 14.

### Patient CSF contains a clonally expanded, H3K27M-reactive B cell population

**Fig. 4.**
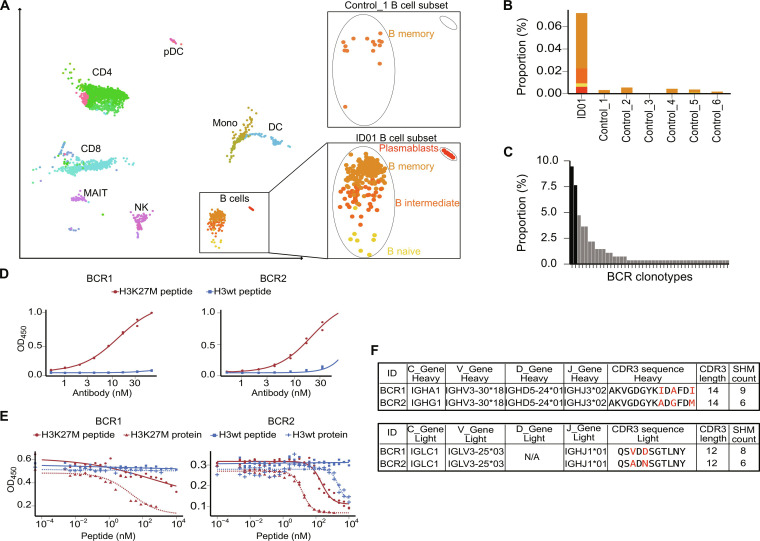
Analysis of the CSF cellular composition of ID01. (**A**) Single-cell sequencing data of ID01 CSF (*n* = 1 sample, *n* = 5316 single cells, left) projected as UMAP. Immune cell clusters were identified by applying Seurat v4 reference mapping. Magnified panels show B cell subpopulations of ID01 compared to a control CSF sample. (**B**) Relative abundance of B cell subclasses in ID01 and control CSF. (**C**) Histogram depicting frequency of identified BCRs in the CSF. Tested BCRs are highlighted in black. (**D**) Dose-response curves of BCRs were assessed using a direct ELISA precoated with either H3wt or H3K27M peptide. OD_450_ was measured and plotted over antibody concentration in nanomolar. Technical duplicates in both panels. (**E**) Affinity to the H3K27M peptide was determined in a competitive ELISA in which the BCRs were incubated with the respective peptide or protein overnight and then added to H3K27M precoated ELISA plates. Dose-response curves show the OD_450_ over peptide concentration in nanomolar. (**F**) Table depicting the VDJ gene usage, CDR3 amino acid sequences, and their acquired somatic hypermutations (SHM) of both chains of BCR1 and BCR2. Highlighted amino acids indicate differences in their CDR3 amino acid sequences.

Having identified tumor-reactive TCRs in the CSF of patient ID01, we compared the composition of this sample to publicly available control datasets (*26*) using Azimuth to map data onto a common reference (*27*). We observed prominent groups of CD19^+^CD20^+^CD27^lo^IgD^−^CD38^−^CD138^−^ memory B cells and CD19^−^CD20^−^CD27^+^IgD^−^CD38^hi^CD138^hi^ plasmablasts in patient ID01 but not in healthy donors (Fig. 4, A and B, and fig. S5). The BCR sequences of these cells showed strong pauciclonality: the five most frequent clones comprise approximately 46% of the overall repertoire (Fig. 4C, *n* = 80 cells). We tested the two most frequent BCRs (9.5 and 7.6%, respectively; Fig. 4C) for their H3K27M binding capacity by producing both as recombinant immunoglobulin G1 (IgG1) antibodies. We used direct enzyme-linked immunosorbent assay (ELISA) to show binding specifically to the H3K27M peptide with similar functional avidities (EC_50_ BCR1 13.1 nM, BCR2 16.5 nM) (Fig. 4D). To determine whether these antibodies bind full-length H3K27M as well as the vaccinating 27-mer H3K27M_p14–40_ peptide, we assessed their affinity in competition with the full-length H3K27M and H3wt proteins. Both antibodies displayed high affinity to the H3K27M protein and the peptide within a range of 10^−7^ and 10^−8^ M, respectively (Fig. 4E). Neither antibody bound appreciably to the H3wt peptide at physiologically relevant concentrations.

Analysis of the heavy and light chain sequences of these two BCRs shows they share a common clonal origin (Fig. 4F). Both receptors exhibit extensive somatic hypermutation, indicating a history of T cell–supported affinity maturation from their germline sequences; furthermore, single-cell transcript data showed that both BCR1 and BCR2 had undergone class-switch recombination to the IgA and IgG1 isotypes, respectively. These results highlight the highly functional CD4^+^ T cell response induced by the peptide vaccine, because cognate B cell differentiation is driven by CD4^+^ T cells recognizing the same epitope (*28*).

## DISCUSSION

In this study, we investigated the immune response of a patient vaccinated with a neoepitope-containing peptide and conducted a comprehensive functional analysis of putative H3K27M-cognate TCRs. We demonstrated that vaccination results in a robust CD4^+^ T cell response leading to a large, polyclonal repertoire of neoantigen-specific TCRs and targeted activation of H3K27M-specific B cells in the CSF.

Furthermore, we demonstrated that the H3K27M peptide vaccine induces an immune response across diverse HLA alleleotypes: The deconvoluted HLA restrictions of cognate CD4^+^ TCRs demonstrate restrictions to members of multiple class II HLA loci. DRB1*13:02 and DQB1*06:03 frequently co-occur on the same haplotype, whereas DRB1*07:01 and DQB1*06 are not a common haplotype pair (*29*). Considering these allelic frequencies and the TCRs described in this work with their restrictions, 40 to 45% of the German population (where the INTERCEPT-H3 trial, NCT04808245, is currently ongoing) could be covered with cell therapy using a pair of identified TCRs. It is notable that many epitope-specific TCRs analyzed here are restricted on the DRB1*13:02 allele, despite this allele:peptide combination not being clearly predicted using common in silico methods. This observation suggests that prediction-based approaches must continue to be supported by functional studies, at a minimum to optimize machine learning or other algorithms used to make such predictions to reduce the number of false negatives in future computational work.

The presence of convergently recombined TCRs (i.e., TCRs that have a shared antigen specificity and identical amino acid sequence but unique nucleotide sequences) has been proposed as a biomarker and indicator for cancer immunotherapy responses (*22*, *30*, *31*)*.* While additional clinical validation of convergent TCRs as a marker is required, it is of great interest that such TCRs (P14, P103, and P2699) are detected in ID01. The use of unique molecular identifiers (UMIs) during reverse transcription, the number of corroborating sequencing reads, and the position of divergent nucleotides directly at the V(D)J recombination junctions strongly suggest that these TCRs are indeed convergent clonotypes. Furthermore, these TCRs are highly abundant and occupy a distinct clonal niche (Fig. 2D), and their relevance is highlighted by their presence within the CSF (Fig. 2E).

We have shown the presence of increasing H3K27M-reactive TCR frequencies in the CSF of patient ID01 upon subsequent vaccine administrations (Fig. 2E). It is notable that T and B cells with neoantigen-cognate receptors would home to the CSF following a subcutaneously administered peptide vaccine unless they detect the endogenous epitope within the central nervous system, presented either by tumor cells or scavenger cells such as macrophages or dendritic cells that have taken up H3K27M-containing debris following tumor cell death. Unfortunately, we were not able to confirm migration into the tumor due to limited sample availability because of the midline and therefore critical localization of these tumors. However, an in-depth study on the immune cell repertoire of matched brain metastases and CSF has shown consistently high clonal overlap of TCRs, suggesting immune cell trafficking between these compartments (*32*). The ability of immune cells to migrate between the CSF and brain lesions was also confirmed in studies focusing on nononcogenic lesions (*33*).

The H3K27M-reactive TCRs in the CSF were identified as central memory CD4^+^ T cells, which have previously been shown to be superior in controlling tumor growth both in vitro and in vivo because of their increased cytokine secretion and prolonged persistence relative to effector T cells (*34*). Moreover, patients successfully treated with immunotherapy maintain highly functional memory T cells years after treatment (*35*). The functionality of the CD4^+^ T cell clones identified in this study is further underlined by the prominent cluster of activated B cells expressing H3K27M-specific BCRs in the CSF. The presence of such tumor-reactive B cells and their intrinsic ability to process and present antigens may support the maintenance of a functional memory CD4^+^ T cell population (*36*). The formation of B cells in tertiary lymphoid structures as well as neoantigen-targeted antibodies have been shown to be correlated with responsiveness to immunotherapy (*37*–*39*). Although not yet explored in detail, genetically engineered B cells carrying tumor-reactive BCRs may comprise another potential immunotherapeutic strategy (*40*). In addition to their beneficial effects on CD4^+^ T cells, they could also indirectly promote tumor killing by activating the complement system upon secretion of neoantigen-specific antibodies, which may recognize HLA-bound neoepitopes in vivo (*40*).

In concordance with previous publications exploring immune responses following neoantigen vaccination (*13*, *18*, *41*), we show that CD4^+^ T cells are the chiefly responding subset in the vaccinated patient ID01. Often overlooked, there is accumulating evidence suggesting that CD4^+^ T cells are at least as critical as cytotoxic CD8^+^ T cells for efficient tumor clearance (*42*, *43*). We have recently published that functional activation of CD4^+^ T cells by glioma-infiltrating myeloid cells is a major determinant of CD8^+^ T cell fitness and prevents their differentiation into terminally exhausted T cells (*44*). In addition, we found this to be critical for responsiveness to immune checkpoint inhibition. Because of the unavailability of a primary tumor cell line for patient ID01, we did not have the opportunity to functionally validate the TCRs in an autologous setting. However, the observed early-onset pseudoprogression, as well as a positive proximity ligation assay, suggest a relevant and functional CD4^+^ T cell response in vivo against the primary tumor presenting the neoepitope H3K27M on HLA-DR molecules.

Few vaccination response studies consider the functional immune receptor repertoire. By performing these studies, it may be possible to learn about the nature of target-specific TCRs, potentially enabling their direct identification by streamlined repertoire analysis in the future (*45*). If no such pattern can be identified, then functional analysis will remain critical, because the sequenced TCR repertoire (even following ex vivo antigen-specific enrichment) is clearly not equivalent to the functional repertoire.

This detailed exploration of the remarkable and long-lasting immune response associated with long-term survival of patient ID01 enabled the identification of TCR and BCR sequences that can both be engineered to create additional cell-based therapies. With the expansion of peptide vaccination to the INTERCEPT-H3 clinical trial cohort, further data will help identify both patient factors leading to responses similar to those described here and individuals most likely to benefit from this precision treatment. Last, the insights gained from characterizing immunotherapeutic responses in DMG are a potent proof of concept for both peptide vaccinations as a therapeutic strategy, as well as leveraging immunogenic T and B cell responses into off-the-shelf, personalized biologic and cellular therapies for further tumor types in the future.

## MATERIALS AND METHODS

### H3K27M vaccination

The 27-mer H3K27M peptide (p14–40) was synthesized by the good manufacturing practice facility of the University of Tübingen, Germany, and 300 μg was emulsified in Montanide (ISA50) at the University Hospital Heidelberg and Mannheim, Germany within 24 hours before vaccination as described previously (*46*). H3K27M was administered subcutaneously with subsequent injection of imiquimod (5% Aldara). Patients received H3K27M-vac after providing written, signed informed consent at the University Hospital of Mannheim, and treatment was approved by the institutional review board.

### PBMC isolation

Heparinized blood from patients was diluted with phosphate-buffered saline (PBS) and layered onto Biocoll Separation Solution (Biochrom) in Leucosep tubes (Greiner Bio-One) followed by a density-gradient centrifugation (800*g* without brake at room temperature). Until further analyses, PBMCs were frozen in 50% freezing medium A [60% X-Vivo 20 and 40% fetal calf serum (FCS)] and 50% medium B [80% FCS and 20% dimethyl sulfoxide (DMSO)] and stored in liquid nitrogen at −140°C.

### IFN-γ ELISpot of PBMC

White-bottom ELISpot HTS plates (MSIPS4W10, Millipore) were hydrophilized with 35% EtOH and subsequently coated with anti-human IFN-γ (1-D1K, Mabtech). Blocking was performed with X-Vivo-20 (Lonza) supplemented with 1% human albumin (Sigma-Aldrich). PBMCs were thawed and rested overnight in X-Vivo-20. Per well, 4 × 10^5^ cells were seeded and stimulated with 2 μg of peptide in a total volume of 100 μl. H3K27M (p14–40), H3wt (p14–40), or MOG (p35–55) was used as a stimulant, and 10% DMSO diluted in aqua ad iniectabilia (Braun) at equal volume was added as negative control. Positive controls were 1-μg staphylococcal enterotoxin B (Sigma-Aldrich), 0.05-μg CMV with 0.05-μg AdV per well, and CEFX Ultra SuperStim Pools of mixed, known peptide epitopes for MHC I– and MHC II–specific stimulation (PM-CEFX-4 and PM-CEFX-3, respectively; JPT Peptide Technologies). After 44 hours, detection of IFN-γ–secreting cells was performed by adding biotinylated anti-human IFN-γ antibodies (7-B6–1), streptavidin-ALP (both Mabtech), and ALP color development buffer (Bio-Rad). IFN-γ Spots were quantified by an ImmunoSpot Analyzer (ImmunoSpot/CTL Europe).

### Peptide-specific T cell expansion

PBMCs were thawed and rested overnight in X-Vivo 20 medium. The next day, cells were resuspended in fresh X-Vivo 20 + 2% human albumin and counted. Half of the available PBMCs were plated in one 24-well plate at 0.5 × 10^6^ cells per 500 μl per well and placed in a 37°C CO_2_ incubator. The remaining cells were plated at the same density in a second 24-well plate but pulsed with (I) 2-μg long H3K27M peptide (p14–40), (II) 2-μg long H3wt peptide (p14–40), or (III) no peptide to control for unspecific expansion, and also placed in the incubator. After 4 hours, unpulsed cells were added to peptide-pulsed wells to a final density of 1 × 10^6^ cells per well.

On days 4, 7, 9, and 11, half of the medium was replaced by 500 μl of fresh medium containing IL-2 (100 IU/ml; Novartis), IL-7 (50 ng/ml; Miltenyi), and IL-15 (50 ng/ml; Miltenyi). On day 14, cells were harvested and rested overnight in cytokine-free media. The next day, cells were subjected to IFN-γ ELISpot analysis as described above to verify the peptide-specific expansion of T cells. A total of 5 × 10^4^ cells were plated per well and restimulated for 44 hours with H3K27M peptide (10 μg/ml), H3wt peptide, left unstimulated or exposed to phorbol 12-myristate 13-acetate (PMA)/ionomycin (20 ng/ml and 1 μg/ml), respectively. Viable cells were cryopreserved for single-cell sequencing.

Expanded cells were also subjected to fluorescence-activated cell sorting (FACS)–based sorting of CD4^+^ and CD8^+^ T cell populations using a BD FACS Aria Fusion I Sorter. Briefly, cells were stained with fixable viability dye (AF700, eBioscience) and then stained with fluorescently labeled antibodies: anti–CD3-BV510 (clone HIT3A, BD), anti–CD4-BV786 (clone SK3 = Leu3a, BD) and anti–CD8a-PerCP/Cy5.5 (clone RPA-T8, Invitrogen). After staining, CD4^+^ and CD8^+^ cell populations were sorted into 0.04% BSA in PBS using a 100-μm nozzle. After FACS, cells were spun down and pellets of CD4^+^ and CD8^+^ enriched cell populations were used for TCRβ deep sequencing.

### TCR β repertoire deep sequencing

For TCR β chain (TCRb) deep sequencing genomic DNA was isolated from PBMC or CD4^+^ and CD8^+^ sorted T cell populations following the DNeasy Blood and Tissue Kit (Qiagen) protocol. Subsequent library preparation was performed by using HsTRBC Kit V7 (Adaptive Biotechnologies) following the manufacturer’s protocol. Libraries were sequenced on an Illumina MiSeq by the Genomics & Proteomics Core Facility, German Cancer Research Center (DKFZ). The provided platform by Adaptive Biotechnologies was used for data processing. Data analysis and visualization was performed using the immunarch 0.6.6 package in RStudio.

### Single-cell RNA and VDJ sequencing

Library construction of the CSF sample was generated using Chromium Single Cell V(D)J Reagent kit v1.1 chemistry (10x Genomics; PN-1000006, PN-1000020, PN-1000005, and PN-120262) following the manufacturer’s protocol. The constructed scVDJ library and scGEX libraries were sequenced on a NovaSeq 6000 platform (Illumina), respectively. Control CSF datasets were retrieved from Gene Expression Omnibus (GEO) with the accession code GSM4104122. Raw sequencing data were processed running cellranger pipeline (version 7.0.0) applying default settings. Subsequent analyses including quality control were performed with Seurat v4 (*47*). The multimodal PBMC reference dataset (*27*) was used to identify cell clusters and was applied using the default settings of FindTransferAnchors() and MapQuery(). The package ScRepertoire v1.5.4 was used to integrate scVDJ and scGEX datasets. pMHC::TCR modeling was performed using ImmuneScape (*23*).

### B cell immortalization

B cell isolation was performed using the the EasySep Human B Cell Isolation Kit (StemCell Technologies). PBMCs were diluted in PBS supplemented with 2% FBS and 1 mM EDTA to a final concentration of 10^7^ cells/ml. Isolated B cells were resuspended in B-LCL-medium containing CpG ODN 2006 (2.5 μg/ml; InvivoGen) and holo-transferrin (30 μg/ml; Sigma-Aldrich). Previously quantified Epstein Barr virus–containing supernatant from B-95 cells was added to the cell suspension before plating 5 × 10^5^ cells per well in a round-bottom 96-well plate.

### Cell culture

B-LCL were cultured in RPMI 1640 medium (Gibco) supplemented with 10% FBS (PAN-Biotech), 1% penicillin-streptomycin (Capricorn Scientific), 50 mM β-mercaptoethanol, 1 mM sodium pyruvate (Thermo Fisher Scientific), and 5 ml of MEM Non-Essential Amino Acids (Gibco). Jurkat T cells (Leibnitz Institute DSMZ #ACC282) were cultured in RPMI 1640 medium supplemented with 10% heat-inactivated FBS and 1% penicillin-streptomycin. HEK293FT (Thermo Fisher Scientific, #R70007) and U-87 MG (ATCC #HTB-14) cells were cultured in Dulbecco’s modified Eagle’s medium (DMEM) (Gibco) supplemented with 10% FBS and 1% penicillin-streptomycin.

### TCR cloning and transgenesis

Sequences for TCR α-/β-variant domains were assembled into a fully human lentiviral TCR-expression vector with TCR α- and β 23 constant domains. TCR-expressing plasmids were obtained from TWIST Biosciences. Lentivirus was produced in human embryonic kidney (HEK) 293T cells in DMEM. HEK cells were transfected with TCR expression vector and lentiviral transfer plasmids using TransIT-VirusGen Transfection Reagent (Mirus #MIR6700) following the manufacturer’s recommendation. Viral supernatant was harvested after 48 hours and sterile filtered with a 0.45 μM syringe filter.

### Coculture assays

B-LCL and TCR-transgenic Jurkat T cells were plated in a 1:3 cell ratio in a round-bottom 96-well plate. U-87 MG cells were treated with IFN-γ (300 IU/ml; Peprotech, #300-02) for 48 hours and plated in a 48-well plate 1 day before adding T cells in a 1:5 ratio. Peptides were added in a final concentration of 10 μM. Cocultures were analyzed after 16 hours of incubation.

### TCR reactivity testing by flow cytometry

For assessing TCR reactivity, cocultures were stained with antibodies targeting surface proteins: anti–CD3-PE/Cy7 (clone HIT3A), anti–CD20-PE/Cy7 (clone 2H7) or anti–CD20-BUV395 (clone L27, BD Biosciences), anti–CD2-PerCP/Cy5.5 (clone RPA-2.10), anti–HA-APC (clone 16B12), and anti–CD69-APC/Cy7 (clone FN50) (all BioLegend). Data were acquired on a BD FACSAria II and a Sony ID7000 Spectral Analyzer. Data analysis for all experiments was performed using FlowJo software v.10.8.1. Determination of TCR reactivity is described in detail in fig. S2.

### Generation of HLA knockout lines

HLA class II knockout lines were generated using the published guide RNA (gRNA) sequences from Lee *et al.* (*48*). The gRNA were ordered as CRISPR RNA and combined in an equimolar ratio with Alt-R CRISPR-Cas9 tracrRNA (IDT). Before electroporation (Neon Transfection system, ThermoFisher Scientific), Alt-R guideRNA duplex was incubated with the Alt-R S.p. HiFi Cas9 Nuclease V3 (IDT) to form a ribonucleoprotein (RNP) complex. Electroporation was performed with Neon 10 μl tips. Briefly, 5 x10^5^ cells were taken up in 9 μl of R-Buffer containing 1 μl of RNP complex and 2 μl of Alt-R Cas9 Electroporation Enhancer (IDT) and electroporated with one pulse at 1400 V for 30 ms. The cells were transferred into 1 ml of B-LCL medium, and expression of HLA-DRA, HLA-DQA, and HLA-DPA was analyzed 6 days later. Negative populations were sorted with a BD FACS Aria II.

### BCR cloning

Sequences for BCR heavy and light chains were assembled into the respective “AbVec” heavy and lambda chain expression vectors (*49*), and prepared as endotoxin-free midipreps using the ZymoPURE II Plasmid Midiprep Kit (Zymo Research).

### Antibody production and purification

Antibodies were produced in HEK293FT cells in DMEM supplemented with 2% FCS. HEK cells were transfected with both heavy and light chain BCR constructs using FuGENE HD transfection reagent (Promega) in a 1:3 ratio. Antibody-containing supernatant was harvested after 5 days and filtered through a 0.45 μM syringe filter. Antibody purification was performed using MabSelect (Cytiva) according to the manufacturer’s guidelines.

### Immunoglobulin G enzyme-linked immunosorbent assay

Indirect ELISA was performed in MaxiSorp plates (Nunc) precoated with human H3K27M and H3wt (p14–40) (180 ng per well in PBS). Purified antibodies were added in serial dilutions.

For performance of the competitive ELISA, 96-well polypropylene plates (Greiner) were blocked with 100% FCS for 2 hours at room temperature. Antibody (125 ng) was diluted with H3K27M and H3wt peptide and protein, respectively and incubated overnight at 4°C. Peptide and proteins were 1:2 serial diluted starting from 144 ng. The following morning the mixture was added to a H3K27M (p14–40, 180 ng) precoated MaxiSorp plate.

Plates were washed with PBS supplemented with 0.025% Tween 20 and blocked with 1X ELISA/ELISpot Diluent (eBioscience). Goat anti-human IgG HRP (1:5000, Southern Biotech) was used as secondary antibody. ELISA signal was developed with tetramethylbenzidine (eBioscience) and stopped with 1 M H_2_SO_4._ Optical density (OD) was measured at 450 nm.

### HLA typing

Genomic DNA was isolated from PBMC of patients or healthy donor controls using the QIAmp DNA Blood Mini Kit (Qiagen) and submitted to DKMZ Germany for high-resolution HLA-typing. For patients, HLA typing was confirmed at the expression level using arcasHLA to analyze single-cell RNA sequencing data (*50*).

## Supplementary Materials

### This PDF file includes:

Figs. S1 to S5 Legend for supplementary excel file

### Other Supplementary Material for this manuscript includes the following:

Supplementary excel file
