# Supplementary material for: H3K27M neoepitope vaccination in diffuse midline glioma induces B and T cell responses across diverse HLA loci of a recovered patient
Source: Sci Adv. 2024 Feb 2;10(5):eadi9091. doi: 10.1126/sciadv.adi9091 (PMC10836722; doi:10.1126/sciadv.adi9091)
Supplement: Supplementary file 1 — Figs. S1 to S5 Legend for supplementary excel file [file sciadv.adi9091_sm.pdf]

Science Advances
AAAS

Supplementary Materials for

H3K27M neoepitope vaccination in diffuse midline glioma induces B and T cell responses across diverse HLA loci of a recovered patient

Tamara Boschert *et al.*

Corresponding author: Edward W. Green, e.green@dkfz.de; John M. Lindner, lindner@bio.mx

Sci. Adv. **10**, eadi9091 (2024)
DOI: 10.1126/sciadv.adi9091

The PDF file includes:

Figs. S1 to S5
Legend for supplementary excel file

Other Supplementary Material for this manuscript includes the following:

Supplementary excel file

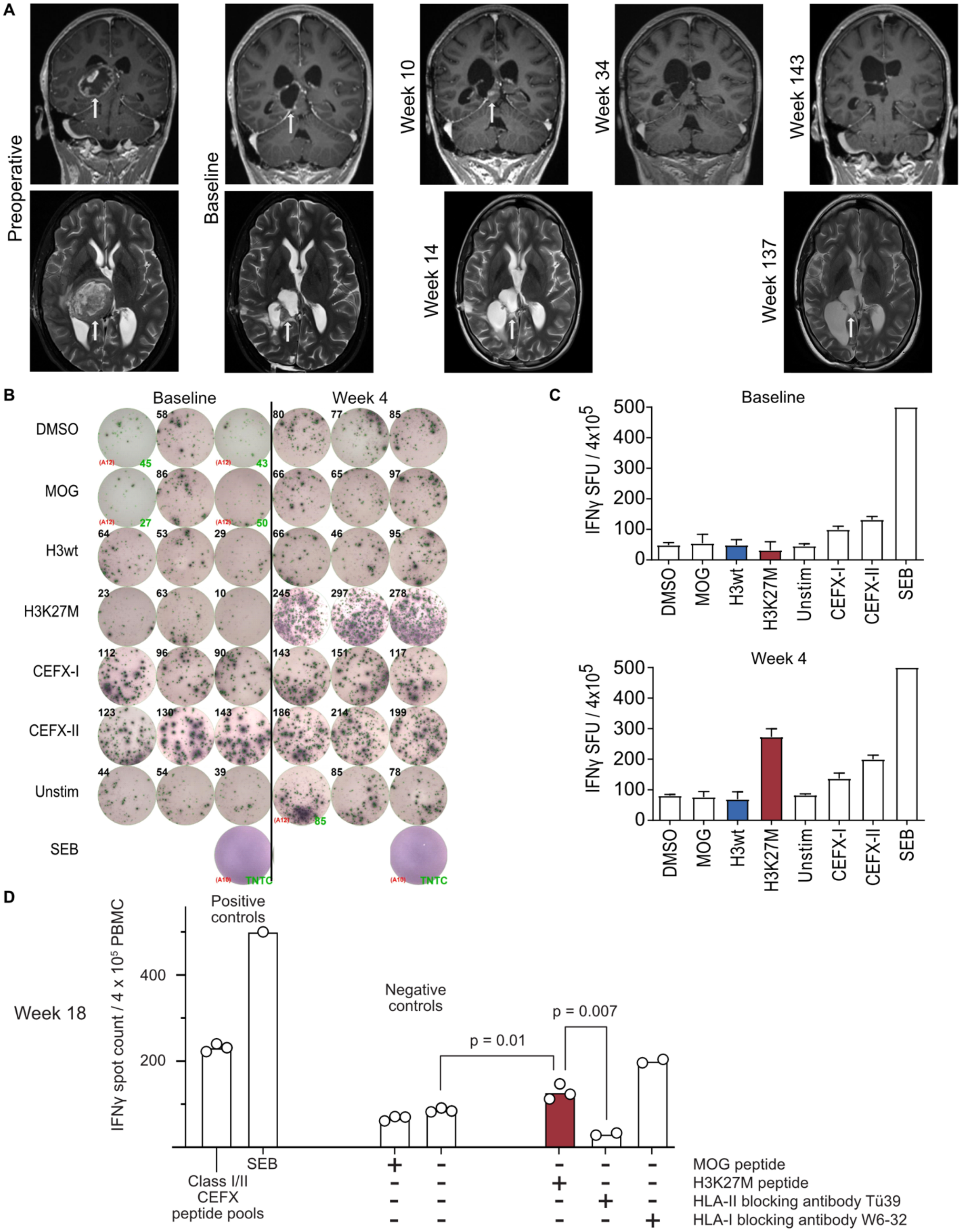

Fig. S1. MRI and ELISpot at baseline and following treatment initiation of patient ID01.

(**A**) T1 weighted with contrast enhancement (top row) and T2 weighted (bottom row) MRI sequences prior to resection, at baseline, and indicated weeks post-treatment initiation. White arrows mark tumor lesion and/or pseudoprogressive disease. (**B**)

Spot counts and (**C**) quantitation of IFNγ ELISpot. DMSO, myelin oligodendrocyte glycoprotein (MOG), and medium only (unstim) serve as negative controls. HLA class I (CEFX-1) and HLA class II (CEFX-II) stimulating peptide pools as well as staphylococcal enterotoxin B (SEB) are used as positive controls. SFU: Spot forming units. (**D**) ELISpot assay at week 18, including HLA blocking antibodies. Statistical significance assessed using one-way ANOVA.

A

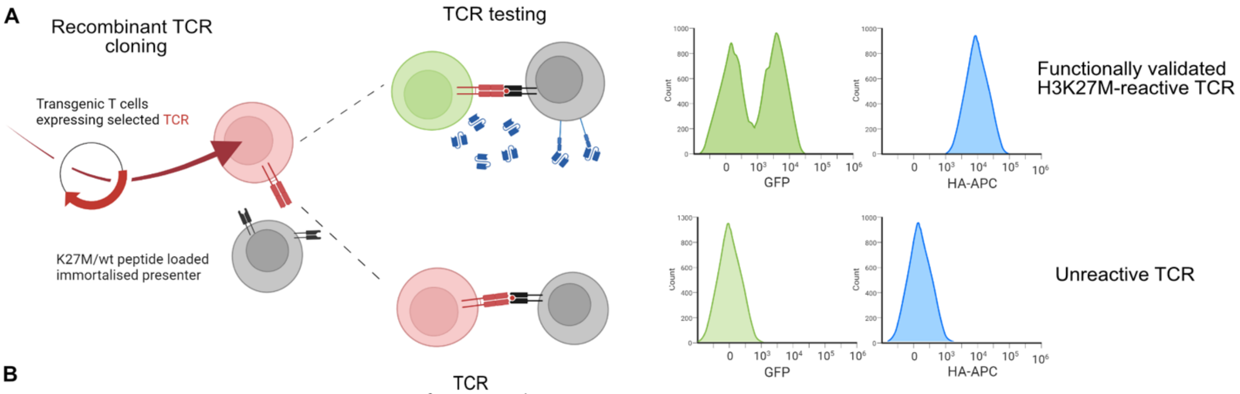

B

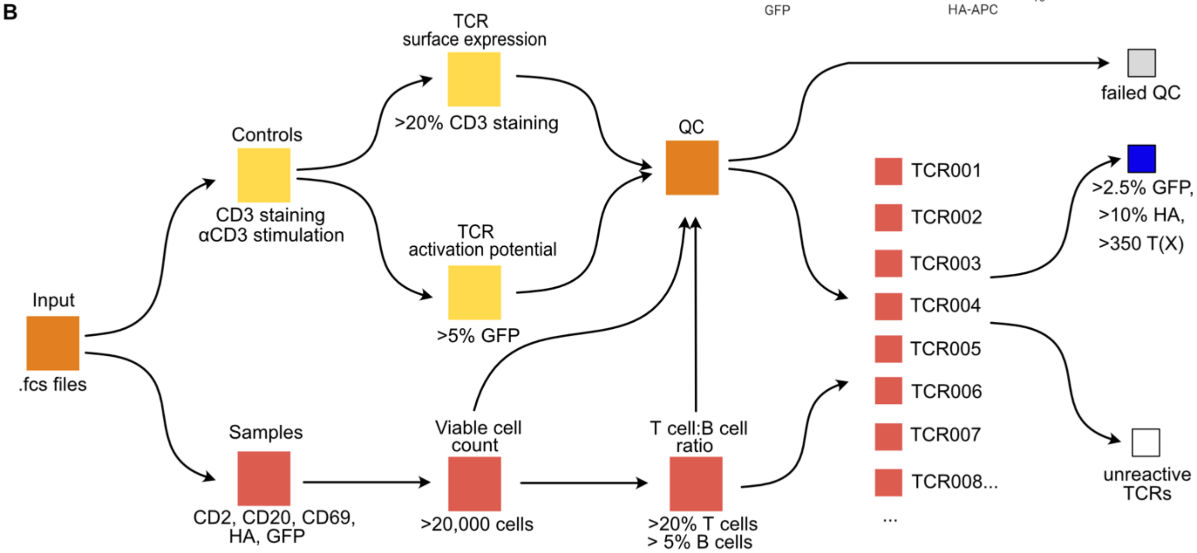

C

TCR	TRBV	CDR3β.aa	TRBJ	TRAV	CDR3α.aa	TRAJ
P1	28	CASTPLQENTEAFF	1-1	12-1	CVVSPNTGGFKTIF	9
C1	14	CASSQEGLEAYNEQFF	2-1	29/DV5	CAASGAGANNLFF	36
P2	7-3	CASSLMTFSAGELFF	2-2	26-2	CIPRNSGNTPLVF	29
P3	11-2	CASSMYLTGTNTEAFF	1-1	8-1	CAVNARYGNNRLAF	7
P7	19	CASSPGQSSQETQYF	2-5	8-6	CAVIRMDSSYKLIF	12
P9	7-2	CASSPGQPNTEAFF	1-1	8-6	CAVTPRGGGNKLTF	10
P13	19	CASSLQGNEAYNEQFF	2-1	8-4	CAVSSNYGGSQGNLIF	42
P14	7-2	CASSLGQANSPLHF	1-6	13-1	CAAKGGFKTIF	9
P16	12-3	CASSFWTNQPQHF	1-5	8-6	CAVSDMGNTPLVF	29
P18	5-1	CASSLSGQGFSGANVLTF	2-6	38-1	CADNDMRF	43
P20	7-2	CASWGGGKLFF	1-4	26-1	CIGTNYGGSQGNLIF	42
P22	4-2	CASSQDGGGAREQYF	2-7	8-4	CAVSDRGNNNDMRF	43
P23	7-3	CASSSGMTGELFF	2-2	8-4	CAVSERGGNKLTF	10
P34	20-1	CSAYRGGLNTEAFF	1-1	13-2	CAETNRKTSYDKVIF	50
P35	2	CASSEPRREETQYF	2-5	9-2	CALSDPGGANSKLTF	56
P36	28	CASRDIGTGVYYEQYF	2-7	12-1	CVVNGDYGQNFVF	26
P38	2	CASRGQAGGRMEAFF	1-1	8-4	CAVSGDSSYKLIF	12
P48	25-1	CASSAGQGMGRNTIYF	1-3	9-2	CALTDGATNKLIF	32
P49	7-2	CASSSGQRNTIYF	1-3	8-4	CALSQRGGSEKLVF	57
P53	2	CASSEGSGANVLTF	2-6	8-4	CAVTPRGNQGGKLIF	23
P58	18	CASSPAWGLEQYF	2-7	38-1	CAFMIGNTGNQFYF	49
P69	7-9	CASSYGQSTEAFF	1-1	8-4	CAVSDRANDYKLSF	20
P72	20-1	CSALADSQETQYF	2-5	8-4	CAVKLAGSYQLTF	28
P82	7-8	CASSLSTAGGEKLFF	1-4	8-4	CAVSDRGYSTLTF	11
P83	3-1	CASSQAQGSSGYTF	1-2	8-4	CAVRPYTGTASKLTF	44
P84	6-6	CASSWDGLETQYF	2-5	36/DV7	CAPYGNQFYF	49
P103	7-2	CASSLGQANSPLHF	1-6	13-1	CAAKGGFKTIF	9
P116	6-2	CASKPTRTGGYGYTF	1-2	36/DV7	CAVGGSEKLVF	57
P1027	27	CASSRGQSDTGELFF	2-2	8-4	CALSDRRSNARLMF	31
P2699	7-2	CASSLGQANSPLHF	1-6	13-1	CAAKGGFKTIF	9
P43b	28	CASSRDRGRSNQPQHF	1-5	8-4	CAVSGTGGTSYGKLTF	52
P52b	6-6	CASSPPGNTIYF	1-3	29/DV5	CAARDNSGGSNYKLTF	53
P6a	7-2	CASSGTGSYTF	1-2	13-1	CAASRTANTDKLIF	34
P77a	19	CASSITVSQPQHF	1-5	35	CAGKPYTSGTYKYIF	40

D

TCR	CDR3β.nt	CDR3α.nt
P14	tgtgccagcagcctaggacaggcaaattcacccctccacttt	tgtgcagcaaagggggggcttcaaaactatcttt
P103	tgtgccagcagcttagggcaagctaattcacccctccacttt	tgtgcagcaaaaggaggcttcaaaactatcttt
P2699	tgtgccagcagcttagggcaagctaattcacccctccacttt	tgtgcagcaaagggggggcttcaaaactatcttt

E

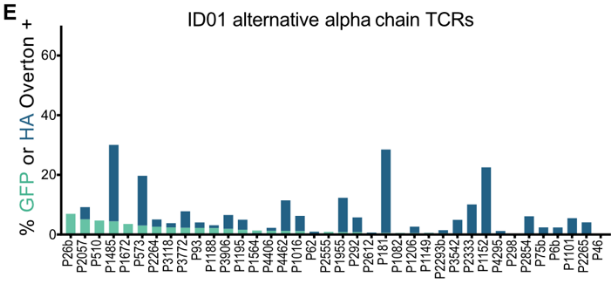

F

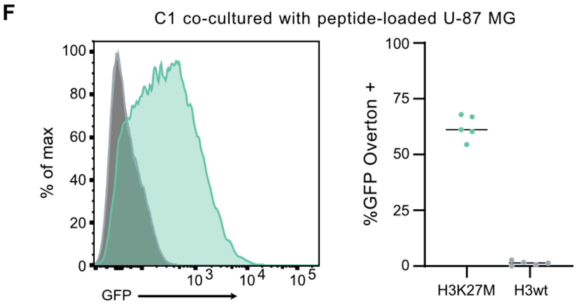

Fig. S2. Screening platform and identification of H3K27M reactive TCRs.

(**A**) Schematic overview of the T-FINDER platform for functional TCR and epitope deconvolution. Selected TCRs are cloned and introduced into the TCR reporter line and subsequently screened for reactivity in co-cultures with peptide-loaded autologous B-LCLs. Reactive TCRs will activate the dual reporter, producing GFP in the T cell line and an αCD19 scFv-mediated anti-HA signal on the antigen-presenting cells (upper panels). (**B**) Workflow for the identification of epitope-cognate TCRs. Each TCR construct is first assessed for CD3 expression levels and αCD3 stimulation potential. Only co-cultures passing the indicated QC thresholds are considered for subsequent analysis. A TCR is considered reactive when both reporter signals surpass the indicated activation cutoffs (blue square). (**C**) List of V and J gene segment usage and CDR3 amino acid sequences of H3K27M-reactive TCRs. Underlined sequences are encoded by the V and J segments, respectively. (**D**) Table highlighting nucleotide differences in the recombination junction sites of the convergently selected TCRs P14, P103, and P2699. (**E**) Bar plot of GFP and anti-HA signals of alternative lower abundance alpha chains paired with the beta chains shown in Fig 2A. (**F**) Representative flow cytometry histograms and quantitation of GFP fluorescence of the H3K27M-reactive TCR C1 co-cultured with peptide-pulsed U87-MG cells (H3K27M in green and H3wt peptide in grey).

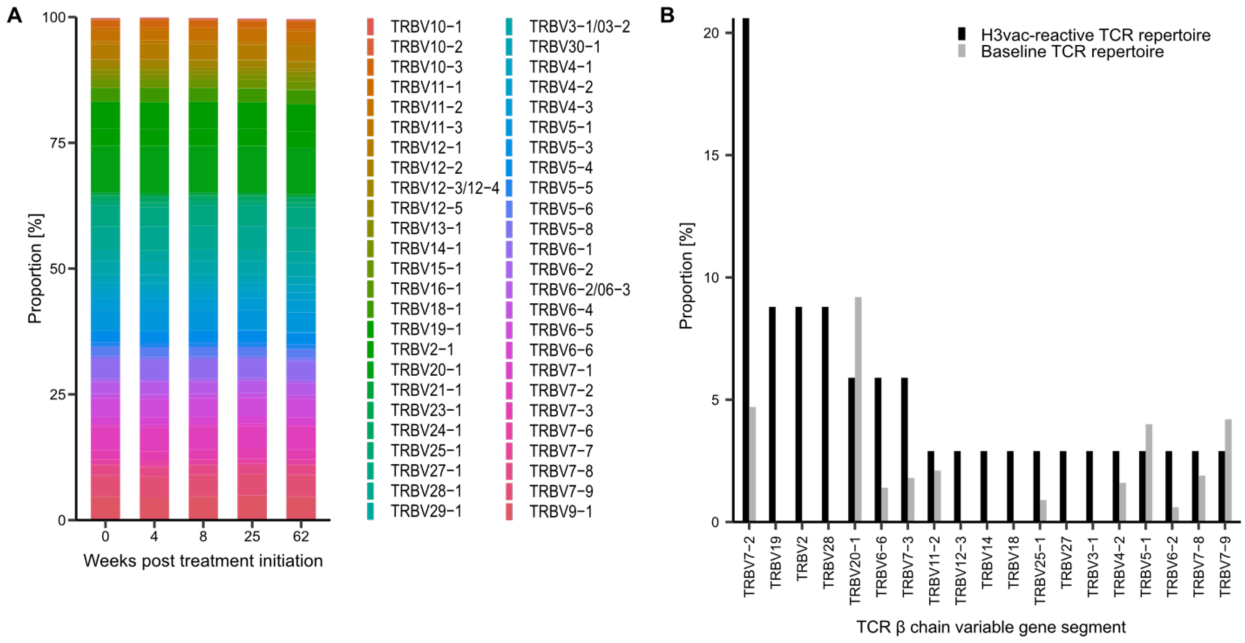

Fig. S3. TRBV gene segment usage in patient ID01.

(**A**) Stacked bar chart showing TRBV gene segment usage and their proportion at indicated weeks. (**B**) Bar charts depicting the proportion of used TRBV gene segments among functionally validated H3K27M-reactive TCRs relative to patient ID01's baseline TCR repertoire.

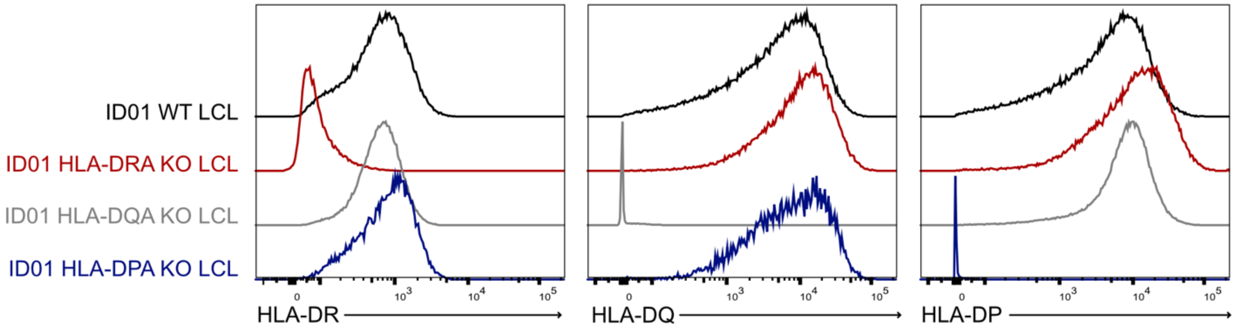

Fig. S4. Validation of CRISPR/Cas9 mediated HLA class II knockouts.

Overlaid histograms depict flow cytometry surface expression analysis of class II HLA complexes (from left to right: DR, DQ, and DP) on patient ID01-derived B-LCLs and of the respective (DR in red, DQ in gray, DP in blue) disrupted alpha chain loci.

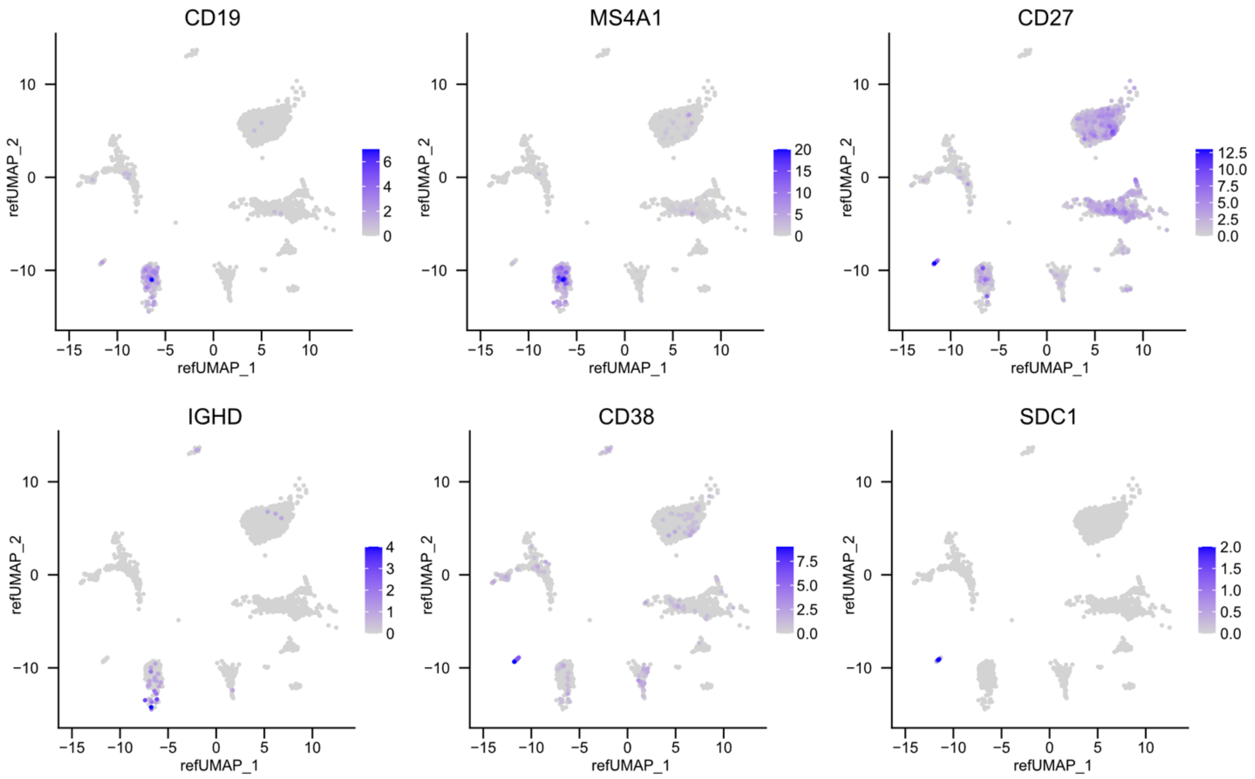

Fig. S5. Gene expression of B cell-specific markers in CSF of ID01.

Feature plots showing patient ID01 cerebrospinal fluid single-cell transcription expression levels of marker genes relevant to the identification of B cell subpopulations.

Supplementary Excel File (Suppl. Excel_seq1_v1.xlsx).

List of T cell receptor sequences and nomenclature used in this study.
